# Supplementary material for: Mushrooms Do Produce Flavonoids: Metabolite Profiling and Transcriptome Analysis of Flavonoid Synthesis in the Medicinal Mushroom Sanghuangporus baumii
Source: J Fungi (Basel). 2022 May 29;8(6):582. doi: 10.3390/jof8060582 (PMC9225156; doi:10.3390/jof8060582)

Journal of *Fungi*

MDPI

Table S1. List of primers used in this study.

Name	Sequence (5′-3′)
PAL-F	TCATCCTCCATTCCATCCCA
PAL-R	TCGCAGCAACTCTTCCTTTT
PAL-B	GCTGATATCGGATCCATGGTCATCAACTCTGACGG
PAL-H	TGCGGCCGCAAGCTTTCACTCCTCAGCGGCAAACA
pAN7-F	AGTAGATGCCGACCGCGGGA
pAN7-R	GGTGATGTCTGCTCAAGCGGG
PAL-iF-F	TGAGCAGACATCACCATGGTCATCAACTCTGACGGTC
PAL-iF-R	CGGTCGGCATCTACTTCACTCCTCAGCGGCAAACAT
hph-F	GAGCCTGACCTATTGCATCT
hph-R	GTCTGCTGCTCCATACAAGC
PAL2-F	TCAGTCCAACATTTGTTGCCA
PAL2-R	GGGAGGTGTGTGCGTTGTAGA
PAL-qF	GCACCCGACGCACTCAAAT
PAL-qR	AAGGCCGTACCGTTCAAAATC
4CL-qF	CCCTTCTGTGCTATTCTTCTGG
4CL-qR	ATTGTTCTATCCTTGCCGTGTC
IFR-qF	TTGCTGTGAAAAAGGGAGGTG
IFR-qR	GAAATGTCAAATCCGAATCGC
PKS-qF	GATGTCCATTCGCCACGGTA
PKS-qR	GAATGCCATAGCACCCTCAGA
α-tubulin-qF	CCAGCAAGCGTTACCGATT
α-tubulin-qR	TCCACGACGTCCATCGTTC

Note: The underlined parts of *PAL*-B and *PAL*-H primers are respectively *Bam*HI and *Hind*III restriction sites.

Table S2. Flavonoids from the mycelia of *S. baumii.*

Compound	Ion mode	Peak area	Formula	Class	CAS
5-Hydroxy-7-methoxyflavone	Negative	7580.90	$C_{16}H_{12}O_4$	Flavones	-
Galangin (3,5,7-Trihydroxyflavone)	Negative	2542.40	$C_{15}H_{10}O_5$	Flavones	548-83-4
6-Hydroxydaidzein	Positive	124290.00	$C_{15}H_{10}O_5$	Isoflavones	17817-31-1
Luteolin (5,7,3',4'-Tetrahydroxyflavone)	Negative	8221.60	$C_{15}H_{10}O_6$	Flavones	491-70-3
3,4,2',4',6'-Pentahydroxychalcone	Positive	45986.00	$C_{15}H_{12}O_6$	Chalcones	73692-51-0
2-Hydroxynaringenin	Negative	11149.00	$C_{15}H_{12}O_6$	Flavones	-
Catechin	Negative	13744.00	$C_{15}H_{14}O_6$	Flavanols	154-23-4
Epicatechin	Positive	30712.00	$C_{15}H_{14}O_6$	Flavanols	490-46-0
4'-Hydroxy-5,7-dimethoxyflavanone	Negative	3749600.00	$C_{17}H_{16}O_5$	Flavanols	-
Taxifolin	Positive	21547.00	$C_{15}H_{12}O_7$	Flavones	480-18-2
Epigallocatechin	Negative	3840.90	$C_{15}H_{14}O_7$	Flavanols	970-74-1
Isobavachalcone D	Positive	34071.00	$C_{19}H_{20}O_4$	Chalcones	-
3-O-Acetylpinobanksin	Negative	3209.30	$C_{17}H_{14}O_6$	Flavanonols	52117-69-8
Quercetagetin	Positive	74531.00	$C_{15}H_{10}O_8$	Flavonols	90-18-6
Gancaonin G	Negative	11933.00	$C_{21}H_{20}O_5$	Isoflavones	126716-34-5
Tangeretin	Positive	5374.80	$C_{20}H_{20}O_7$	Flavonols	481-53-8
Nobiletin	Positive	7655.10	$C_{21}H_{22}O_8$	Flavones	478-01-3
Apigenin-7-O-glucoside	Positive	8730.20	$C_{21}H_{20}O_{10}$	Flavones	578-74-5
Genistein-7-O-galactoside	Positive	7158.10	$C_{21}H_{20}O_{10}$	Isoflavones	-
Apigenin-7-O-α-D-glucopyranoside	Positive	9012.50	$C_{21}H_{20}O_{10}$	Flavones	-
Apigenin-3'-O-α-D-glucopyranoside	Positive	11641.00	$C_{21}H_{20}O_{10}$	Flavones	-
7-O-Galloyltricetiflavan	Negative	13607.00	$C_{22}H_{18}O_{10}$	Flavanols	-
Epicatechin gallate	Negative	14254.00	$C_{22}H_{18}O_{10}$	Flavanols	1257-08-5
Catechin gallate	Negative	13965.00	$C_{22}H_{18}O_{10}$	Flavanols	130405-40-2
Prunetin-4'-O-glucoside	Negative	460880.00	$C_{22}H_{22}O_{10}$	Isoflavones	154-36-9
Trifolirhizin	Negative	139630.00	$C_{22}H_{22}O_{10}$	Isoflavanone	6807-83-6
Kaempferol-7-O-glucoside	Negative	20634.00	$C_{21}H_{20}O_{11}$	Flavonols	16290-07-6
Trifolin	Negative	21323.00	$C_{21}H_{20}O_{11}$	Flavonols	23627-87-4
Dihydrokaempferol-3-O-glucoside	Positive	5569.80	$C_{21}H_{22}O_{11}$	Flavanonols	1049-08-8

Compound	Ion mode	Peak area	Formula	Class	CAS
Epigallocatechin-3-gallate	Negative	24306.00	$C_{22}H_{18}O_{11}$	Flavanols	989-51-5
Gallocatechin 3-O-gallate	Negative	21262.00	$C_{22}H_{18}O_{11}$	Flavanols	5127-64-0
(-)-Gallocatechin gallate	Positive	21819.00	$C_{22}H_{18}O_{11}$	Flavones	4233-96-9
Kaempferol-3-O-glucuronide	Negative	5549.50	$C_{21}H_{18}O_{12}$	Flavonols	22688-78-4
Luteolin 7-O-glucuronide	Positive	4403.00	$C_{21}H_{18}O_{12}$	Flavones	29741-10-4
8-Methoxykaempferol-7-O-rhamnoside	Negative	4431.60	$C_{22}H_{22}O_{11}$	Flavonols	-
Quercetin-7-O-glucoside	Negative	8272.30	$C_{21}H_{20}O_{12}$	Flavonols	491-50-9
Isoquercitrin	Negative	6347.10	$C_{21}H_{20}O_{12}$	Flavonols	482-35-9
Hyperin	Negative	8540.20	$C_{21}H_{20}O_{12}$	Flavonols	482-36-0
Spiraeoside	Negative	13059.00	$C_{21}H_{20}O_{12}$	Flavonols	20229-56-5
Hesperetin-7-O-glucoside	Positive	16419.00	$C_{22}H_{24}O_{11}$	Flavones	31712-49-9
Hesperetin-5-O-glucoside	Negative	11927.00	$C_{22}H_{24}O_{11}$	Flavanonols	69651-80-5
Apigenin-7-O-(6"-acetyl) glucoside	Positive	28380.00	$C_{23}H_{22}O_{11}$	Flavones	72741-92-5
Kaempferol-3-O-(4"-O-acetyl) rhamnoside	Positive	35057.00	$C_{23}H_{22}O_{11}$	Flavones	-
6-C-Methylquercetin-3-O-glucoside	Positive	3144.00	$C_{22}H_{22}O_{12}$	Flavonols	-
6"-O-Acetylglycitin	Positive	557220.00	$C_{24}H_{24}O_{11}$	Isoflavones	134859-96-4
Kaempferol-3-O-(6"-acetyl) glucoside	Negative	952530.00	$C_{23}H_{22}O_{12}$	Flavonols	-
Kaempferol-3-O-(2"-acetyl) glucoside	Positive	3674500.00	$C_{23}H_{22}O_{12}$	Flavonols	-
Quercetin-3-O-(2"-acetyl) rhamnoside	Positive	7592.90	$C_{23}H_{22}O_{12}$	Flavonols	-
Tricin-5-O-Glucoside	Negative	12845.00	$C_{23}H_{24}O_{12}$	Flavones	32769-00-9
6"-O-Malonyldaidzin	Positive	215770.00	$C_{24}H_{22}O_{12}$	Isoflavones	124590-31-4
Kaempferol-3-O-(2"-O-acetyl) glucuronide	Negative	106010.00	$C_{23}H_{20}O_{13}$	Flavonols	-
6"-O-Malonylgenistin	Positive	31107.00	$C_{24}H_{22}O_{13}$	Isoflavones	51011-05-3
Naringenin-7-O-(6"-malonyl) glucoside	Positive	3296.10	$C_{24}H_{24}O_{13}$	Flavanols	-
6"-O-Malonylglycitin	Positive	6589.20	$C_{25}H_{24}O_{13}$	Isoflavones	137705-39-6
Procyanidin B1	Negative	30624.00	$C_{30}H_{26}O_{12}$	Proanthocyanidins	20315-25-7
Procyanidin B3	Negative	2411.70	$C_{30}H_{26}O_{12}$	Proanthocyanidins	23567-23-9
Procyanidin B2	Negative	13885.00	$C_{30}H_{26}O_{12}$	Proanthocyanidins	29106-49-8
Apigenin-7-O-(6"-p-Coumaryl) glucoside	Positive	14834.00	$C_{30}H_{26}O_{12}$	Flavones	-
Isorhoifolin	Positive	5643.00	$C_{27}H_{30}O_{14}$	Flavones	552-57-8
Rhoifolin	Positive	7548.50	$C_{27}H_{30}O_{14}$	Flavones	17306-46-6

Compound	Ion mode	Peak area	Formula	Class	CAS
Narirutin	Negative	6377.30	$C_{27}H_{32}O_{14}$	Flavanones	14259-46-2
Naringin	Negative	23435.00	$C_{27}H_{32}O_{14}$	Flavanones	10236-47-2
Kaempferol-3-O-(2"-p-Coumaroyl) galactoside	Positive	25450.00	$C_{30}H_{26}O_{13}$	Flavones	-
Kaempferol-3-O-(6"-p-Coumaroyl) galactoside	Positive	11176.00	$C_{30}H_{26}O_{13}$	Flavones	-
Tiliroside	Negative	7414.40	$C_{30}H_{26}O_{13}$	Flavones	20316-62-5
Castanoside A [Kaempferol-3-O-(6"-p-coumaroyl) mannoside]	Positive	13934.00	$C_{30}H_{26}O_{13}$	Flavones	-
Luteolin-7-O-(6"-caffeoyl) rhamnoside	Negative	1123.00	$C_{30}H_{26}O_{13}$	Flavones	-
Kaempferol-3-O-neohesperidoside	Positive	9562.10	$C_{27}H_{30}O_{15}$	Flavones	32602-81-6
Kaempferol-3-O-rhamnosyl (1→2) glucoside	Positive	9246.00	$C_{27}H_{30}O_{15}$	Flavonols	-
Rutin	Negative	12282.00	$C_{27}H_{30}O_{16}$	Flavonols	153-18-4
Quercetin-3-O-glucoside-7-O-rhamnoside	Positive	7419.00	$C_{27}H_{30}O_{16}$	Flavonols	-
Quercetin-3-O-robinobioside	Negative	6797.50	$C_{27}H_{30}O_{16}$	Flavonols	52525-35-6
Luteolin-7,3'-di-O-glucoside	Positive	7557.50	$C_{27}H_{30}O_{16}$	Flavones	52187-80-1
Quercetin-3-O-(4"-O-glucosyl) rhamnoside	Positive	8766.80	$C_{27}H_{30}O_{16}$	Flavonols	59262-54-3
Quercetin-3-O-neohesperidoside	Positive	11836.00	$C_{27}H_{30}O_{16}$	Flavonols	117611-67-3
Guangsangon N	Positive	2782.80	$C_{35}H_{30}O_{10}$	Flavanones	-
Neohesperidin	Positive	7706.40	$C_{28}H_{34}O_{15}$	Flavanones	13241-33-3
Hesperidin	Negative	25349.00	$C_{28}H_{34}O_{15}$	Flavanones	520-26-3
Tamarixetin-3-O-glucoside-7-O-rhamnoside	Positive	60425.00	$C_{28}H_{32}O_{16}$	Flavones	-
Kaempferol-3-O-(2-O-Xylosyl-6-O-Rhamnosyl) Glucoside	Positive	4424.00	$C_{32}H_{38}O_{19}$	Flavones	-
Kaempferol-3-O-rutinoside-7-O-glucoside	Positive	59905.00	$C_{33}H_{40}O_{20}$	Flavonols	-

Table S3. Homologous amino-acid sequences for PAL from 16 species.

Species	Accession ID	Kingdom
Tricholoma matsutake	BAM63315.2	Agaricales; Tricholomataceae
Pleurotus eryngii	AHH55203.1	Agaricales; Pleurotaceae
Flammulina velutipes	AHD25301.1	Agaricales; Physalacriaceae
Sparassis crispa	XP 027611961.1	Polyporales; Sparassidaceae
Daedalea quercina	KZT72322.1	Polyporales
Laetiporus sulphureus	XP 040759197.1	Polyporales
Cantharellus anzutake	XP 038919162.1	Cantharellales; Cantharellaceae
Pyrrhoderma noxium	PAV24234.1	Hymenochaetales; Hymenochaetaceae
Fomitiporia mediterranea	XP 007262022.1	Hymenochaetales; Hymenochaetaceae
Sanghuangporus baumii		Hymenochaetales; Hymenochaetaceae
Inonotus obliquus	AJD20419.1	Hymenochaetales; Hymenochaetaceae
Aspergillus fumigatus	KEY77231.1	Eurotiales; Aspergillaceae
Fusarium langsethiae	KPA41552.1	Hypocreales; Nectriaceae
Aspergillus luchuensis	GAT31350.1	Eurotiales; Aspergillaceae
Penicillium griseofulvum	XP 040645987.1	Eurotiales; Aspergillaceae
Arabidopsis thaliana	NP 187645.1	Brassicales; Brassicaceae

Figure S1. Micro-array plot of differentially expressed genes.

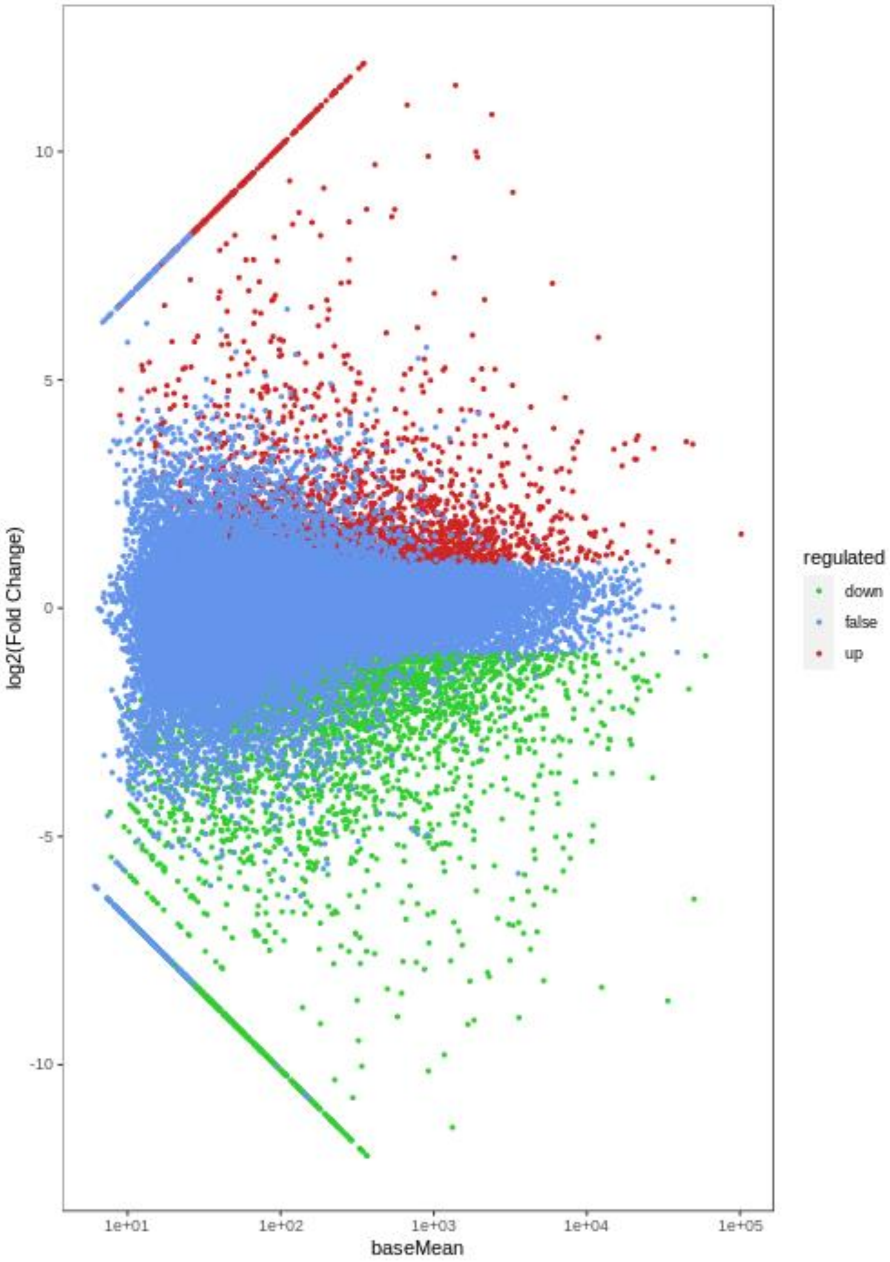

Figure S2. The SbPAL 2,493 bp open reading frame and encoded amino acid sequence.

```
1     ATGGTCATCAACTCTGACGGTCACTTCGCGCGTCCTCATTCAAACTCAAACTCGGGTTCCGCGACTACAACCATC
       M  V  I  N  S  D  G  H  F  A  R  P  H  S  N  S  N  S  G  S  A  T  T  T  I
76    GTCTCCCCTCACATCGACCACTCTTACGACAGCTACTATGGTCATGCTATCCCCCTTCGTGTACCAACACCGGAG
       V  S  P  H  I  D  H  S  Y  D  S  Y  Y  G  H  A  I  P  L  R  V  P  T  P  E
151   GACTTGCTCAAGCACAAGGCACTCCTGAACCAAGCAGACTGTCTCAAAGACTTCATCGCGTCCTACCAGGAGCTC
       D  L  L  K  H  K  A  L  L  N  Q  A  D  C  L  K  D  F  I  A  S  Y  Q  E  L
226   GACTCGTACAAGAACGGCGCTTCCATCAGCCTTACAGGTGATGATCTCACCCTCTCTGCAGTCGTAGCCGTCGCA
       D  S  Y  K  N  G  A  S  I  S  L  T  G  D  D  L  T  L  S  A  V  V  A  V  A
301   CGCTACCCAGAGCTCGTCTCTGCAGCTATCGACGAAGCTCTCGCGTCCCTCTCCAAGGACGACTCGCGCGTCCAG
       R  Y  P  E  L  V  S  A  A  I  D  E  A  L  A  S  L  S  K  D  D  S  R  V  Q
376   GCTCTCAACAGCTCACGAAAGATCATCGATGACAAGCTCGCGCAGAATAAGAGTATCTATGGTGTCAGCACCGGT
       A  L  N  S  S  R  K  I  I  D  D  K  L  A  Q  N  K  S  I  Y  G  V  S  T  G
451   TTCGGTGGTAGTGCGGACACTCGTACGGCAGAGCACGATCTACTCGGTTTCGCGCTCCTTCAGCACCAGCATTCG
       F  G  G  S  A  D  T  R  T  A  E  H  D  L  L  G  F  A  L  L  Q  H  Q  H  S
526   GGTGTCCTCCCGACTGGTCTCCAGTCCGTCGCGGATGCCAATGCTAGCTCTGACGCACCGTCCTCTGCTGCTTCG
       G  V  L  P  T  G  L  Q  S  V  A  D  A  N  A  S  S  D  A  P  S  S  A  A  S
601   ACTACCAAGTCGAGCACTCCCCTCCCGTTGAGCTCTTCCGCTTCCTCCCTCTCCATGCCCGCCGCATGGACGCGT
       T  T  K  S  S  T  P  L  P  L  S  S  S  A  S  S  L  S  M  P  A  A  W  T  R
676   GCAGCCATGGTAGTTCGTCTCAACTCCCTCCTGCGGGGACACAGTGCTGCTTCTGTTCCCCTCCTCAACGCCATG
       A  A  M  V  V  R  L  N  S  L  L  R  G  H  S  A  A  S  V  P  L  L  N  A  M
751   GGCGGACTTCTCTCCAAGAATATCACGCCTATCGTCCCTCTCCGTGGTTCCATCTCCGCTTCAGGCGACTTGTCT
       G  G  L  L  S  K  N  I  T  P  I  V  P  L  R  G  S  I  S  A  S  G  D  L  S
826   CCTCTTTCTTACGTCGCCGGTACGCTCGTCGGCGAGCGAGGCATCTACTGCTACGCACCTAGCGGTGAGAAGCGC
       P  L  S  Y  V  A  G  T  L  V  G  E  R  G  I  Y  C  Y  A  P  S  G  E  K  R
901   CTAGGTGAGGAACTGGGTATGAGCGGAGAGGGAACGAAGGTCCTCCGTGCACCCGACGCACTCAAATCTGCTGGT
       L  G  E  E  L  G  M  S  G  E  G  T  K  V  L  R  A  P  D  A  L  K  S  A  G
976   CTTGAGCCCATCAAGCTCAGACCAAAGGAGCAGCTAGCGATTTTGAACGGTACGGCCTTCAGTTGTGGAGCAGCT
       L  E  P  I  K  L  R  P  K  E  Q  L  A  I  L  N  G  T  A  F  S  C  G  A  A
1051  GCTCTCTGCGTCGAGGAGGCGAGGCAACTCGTCATGCTCGGGACGGTCTGCACCGCAATGGGCACAGAGGCCATG
       A  L  C  V  E  E  A  R  Q  L  V  M  L  G  T  V  C  T  A  M  G  T  E  A  M
1126  CGTGGCTCAGCAGACTCCTTCTGCGAGTTCATCCAGCGCATACGTCCGCACCCGGGGCAGATCGAGACAGGCGCG
       R  G  S  A  D  S  F  C  E  F  I  Q  R  I  R  P  H  P  G  Q  I  E  T  G  A
1201  CTCCTCACACACTTGTTGGAGACCTCGAAGCTCGCTACACGCCATGCTGAGCCCGGACACTCTGCCGCGGTCAAT
       L  L  T  H  L  L  E  T  S  K  L  A  T  R  H  A  E  P  G  H  S  A  A  V  N
1276  GGCGTTGAGGAGAGCATGACGGAGACCGGGAAGGATAAGATCATTGTTGATGAGGAAACTATTGATGCGGATGCC
       G  V  E  E  S  M  T  E  T  G  K  D  K  I  I  V  D  E  E  T  I  D  A  D  A
1351  GGTGTCTTGAGGCAGGATCGTTACCCACTCAGGACCGCCCCGCAATGGCTCGGTCCGCAACTTGAGACCGTCCAG
       G  V  L  R  Q  D  R  Y  P  L  R  T  A  P  Q  W  L  G  P  Q  L  E  T  V  Q
1426  CGTGCTGCTGAAGTAATTGCTATCGAGTGCAATTCTACGACGGATAACCCACTCATCGACCCTGCAACTGGTATT
       R  A  A  E  V  I  A  I  E  C  N  S  T  T  D  N  P  L  I  D  P  A  T  G  I
1501  GTCCACCACGGCGGTAACTTCCAAGCAATGGCCGTCACCTCCGTCCTCGAACCACTCCGTCTCTCCCTCTTCCAC
       V  H  H  G  G  N  F  Q  A  M  A  V  T  S  V  L  E  P  L  R  L  S  L  F  H
1576  ATCGGCAAGATCCTCTTCGCGCAAGCGACCGAGCTGCAGAACCCTCTCATGTCGAATGGGCTGACGGGTAACCTC
       I  G  K  I  L  F  A  Q  A  T  E  L  Q  N  P  L  M  S  N  G  L  T  G  N  L
1651  GCCTCCACCGACGCGTCCCTCAACTTCGCCGGTAAGGGGATCGACATCGCGATGGCCGCGTACGTCGCCGAGCTC
       A  S  T  D  A  S  L  N  F  A  G  K  G  I  D  I  A  M  A  A  Y  V  A  E  L
1726  GCGTACCTTGCGAACCCGGTGAGTACGGGCGTGCAGAGCGCCGAGATGCACAACCAGGCTGTGAACTCGTTGGCA
       A  Y  L  A  N  P  V  S  T  G  V  Q  S  A  E  M  H  N  Q  A  V  N  S  L  A
1801  TTCGTCAGCGCGCGGTACACCCTCCAGGCGATTGAGATCGTGCAGATGGTTGTTGCGAGCTACATCTACCTCCTC
       F  V  S  A  R  Y  T  L  Q  A  I  E  I  V  Q  M  V  V  A  S  Y  I  Y  L  L
1876  TGCCAGGCTGTCGACCTCCGCGCCCTGCAGAAGGAGATGGAAGAGAAGACGCAGGAGATCGTCGAGGAACTTGTT
       C  Q  A  V  D  L  R  A  L  Q  K  E  M  E  E  K  T  Q  E  I  V  E  E  L  V
1951  AAGGAGCACTTTGCTGCTGGTGAAAATTCCGTCAATGCGAAGGAAGTGTCCAAGGCTGTTTGGACGTCATTCGAC
       K  E  H  F  A  A  G  E  N  S  V  N  A  K  E  V  S  K  A  V  W  T  S  F  D
2026  ACCTCTGCAAACATGGATGCGAAGCCCCGTGCTGAGAAGGCCGCGAAGGCATCTACGCAGCCTCTCGTAGACCAG
       T  S  A  N  M  D  A  K  P  R  A  E  K  A  A  K  A  S  T  Q  P  L  V  D  Q
2101  CTCCTCTCCTCCCACAGCGTCTCCTTCTCACTTTCATCCCTCCCCGCCTTCCAATCGAGCCTCTCCGCGCGTATC
       L  L  S  S  H  S  V  S  F  S  L  S  S  L  P  A  F  Q  S  S  L  S  A  R  I
2176  TACAACGCACACACCTCCCTCACCACCTCCTACCTCTCTTCCACCGCTTCCCCGTCCTCGCCTGCGTACCTCCCA
       Y  N  A  H  T  S  L  T  T  S  Y  L  S  S  T  A  S  P  S  S  P  A  Y  L  P
2251  GCCCTCCAGCTCCTCGGGCGCACCAAGTCCCTCTACGCGTTCGTCCGCTCGCCCGTCTCCCAGGGTGGGTTAGGC
       A  L  Q  L  L  G  R  T  K  S  L  Y  A  F  V  R  S  P  V  S  Q  G  G  L  G
2326  ATCGGCATGCACGGATACGAGAACCTCCAGAAATTCGTCGACGGGCTCGGGAACGGCGACGCTAGCAAGCGTGGC
       I  G  M  H  G  Y  E  N  L  Q  K  F  V  D  G  L  G  N  G  D  A  S  K  R  G
2401  ACGATCGGTGGGGACGTCAGCGCGATCTACGAGAGCATTCGCGATGGACGCATGGGCCGCGTCGTCGTTAGCATG
       T  I  G  G  D  V  S  A  I  Y  E  S  I  R  D  G  R  M  G  R  V  V  V  S  M
2476  TTTGCCGCTGAGGAGTGA
       F  A  A  E  E  *
```

Figure S3. PCR gel electrophoresis results for the resistant strains (M: marker; N: negative control; P: positive control; 1, 2: Band for the hph gene; 3, 4: Band for the PAL gene initiated by a gpd promoter).

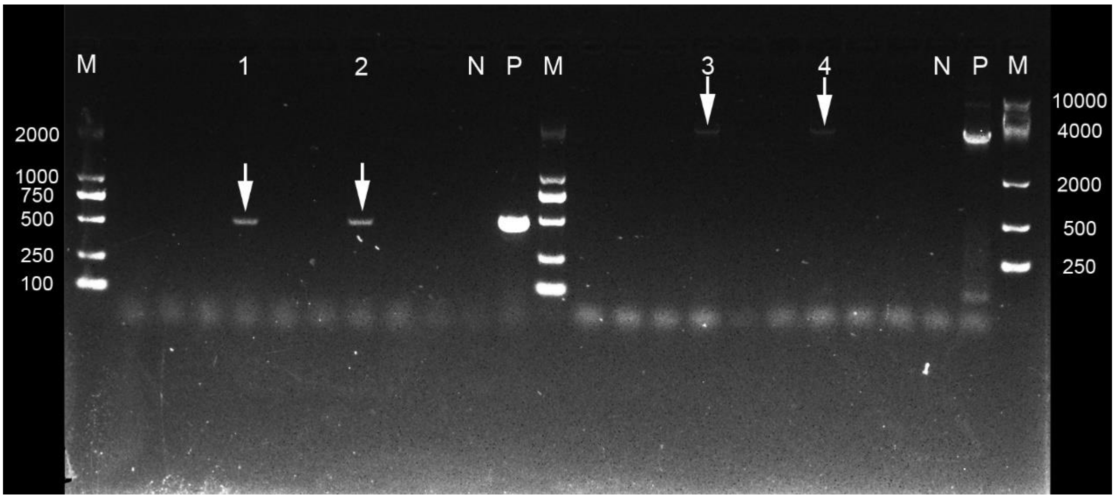

Supplement: Supplementary file 1 [file jof-08-00582-s001.zip › jof-1718418-supplementary-done.pdf]
